# RNA Solutions: Synthesizing Information to Support Transcriptomics (RNASSIST)

**DOI:** 10.1093/bioinformatics/btab673

**Published:** 2021-09-27

**Authors:** Yi-Pei Chen, Laura B Ferguson, Nihal A Salem, George Zheng, R Dayne Mayfield, Mohammed Eslami

**Affiliations:** Netrias, LLC, Annapolis, MD 21409, USA; Department of Neuroscience, The Waggoner Center for Alcohol and Addiction Research, The University of Texas at Austin, Austin, TX 78712, USA; Department of Neuroscience, The Waggoner Center for Alcohol and Addiction Research, The University of Texas at Austin, Austin, TX 78712, USA; Netrias, LLC, Annapolis, MD 21409, USA; Department of Neuroscience, The Waggoner Center for Alcohol and Addiction Research, The University of Texas at Austin, Austin, TX 78712, USA; Netrias, LLC, Annapolis, MD 21409, USA

## Abstract

**Motivation:**

Transcriptomics is a common approach to identify changes in gene expression induced by a disease state. Standard transcriptomic analyses consider differentially expressed genes (DEGs) as indicative of disease states so only a few genes would be treated as signals when the effect size is small, such as in brain tissue. For tissue with small effect sizes, if the DEGs do not belong to a pathway known to be involved in the disease, there would be little left in the transcriptome for researchers to follow up with.

**Results:**

We developed RNA Solutions: Synthesizing Information to Support Transcriptomics (RNASSIST), a new approach to identify hidden signals in transcriptomic data by linking differential expression and co-expression networks using machine learning. We applied our approach to RNA-seq data of post-mortem brains that compared the Alcohol Use Disorder (AUD) group with the control group. Many of the candidate genes are not differentially expressed so would likely be ignored by standard transcriptomic analysis pipelines. Through multiple validation strategies, we concluded that these RNASSIST-identified genes likely play a significant role in AUD.

**Availability and implementation:**

The RNASSIST algorithm is available at https://github.com/netrias/rnassist and both the software and the data used in RNASSIST are available at https://figshare.com/articles/software/RNAssist_Software_and_Data/16617250.

**Supplementary information:**

Supplementary data are available at *Bioinformatics* online.

## 1 Introduction

Transcriptomic analysis is a powerful tool to quantify changes in gene expression induced by treatments or disease states. The results of a transcriptome can be used to develop molecular signatures or biomarkers of diseases (Han and Jiang, 2014; Hong *et al.*, 2020). A standard transcriptomic analysis workflow consists of quantification of transcripts and computing differential expression between test and control conditions (Conesa *et al.*, 2016). A cutoff for fold change and statistical significance values are then used to find differentially expressed genes (DEGs). By design, the threshold for DEGs is a moving target. |log_2_(fold change)| > 2 and adjusted *P*-values < 0.05 as the threshold is commonly used but there is no strict rule against a different cutoff (Eswaran *et al.*, 2012; Guo *et al.*, 2013; Kaczkowski *et al.*, 2016). To then interpret the results, functional profiling, such as pathway analysis, is performed on DEGs (Conesa *et al.*, 2016). For well-annotated genes, a DEG is considered relevant if previous literature supports its role in a function related to the disease under investigation.

The challenge with the standard transcriptomic analysis is that not all the transcriptomic data have similar effect sizes. For example, the largest size of the differential expression for substance abuse and psychiatric disorders in brain could be as small as |log_2_(fold change)| < 1 while for diseases like cancers, |log_2_(fold change)| could be as large as 10 or more (Eswaran *et al.*, 2012; Guo *et al.*, 2013; Huggett and Stallings, 2020; Kaczkowski *et al.*, 2016; Ramaker *et al.*, 2017). For transcriptomes with small effect sizes, many potentially important transcripts would be erroneously considered as noise and filtered out by standard analyses since they have no clear link to the disease. This process ignores the genes that regulate or are regulated by the disease which are not differentially expressed. Furthermore, even if a gene is differentially expressed, if it does not belong to a biological pathway known to play a role in the disease, it is likely ignored. Many biological pathways are annotated with general terms such as kinase signaling or DNA replication and have not been linked to specific disease states. Unless researchers have prior knowledge of the involvement of these pathways, it is not trivial to elucidate their roles in diseases. New techniques and functional validation are thus required to discover the underlying structure in the transcriptomic data that can link to disease phenotype.

**Fig. 1.**
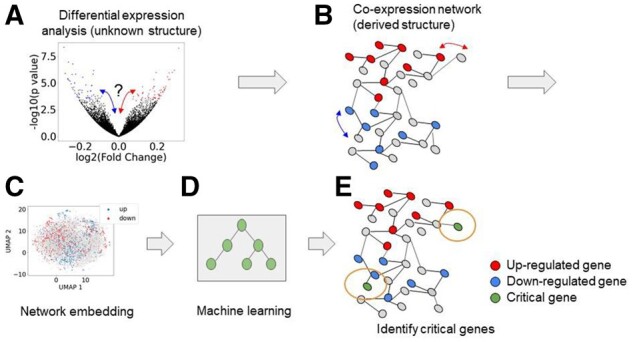
Motivation for the development of RNASSIST. State-of-the-art approaches can extract transcriptional signals from noise through techniques such as differential expression and co-expression network analyses (**A and B**). The challenge with gene expression data is that the effect size is small in tissues like the brain. This leads to most of the transcripts to be in the noise (black dots in A) with no clear link to the disease phenotype. Techniques such as co-expression network analysis (B) provide structure to transcriptomic data that can link non-DEGs to DEGs. Here, we present a technique that uses network embedding (**C**) and machine learning (**D**) to integrate network structure with DEGs to identify critical genes that are relevant to disease phenotypes where the majority of them are not differentially expressed (**E**)

In this article, we describe a new approach to analyze transcriptomics by developing RNA Solutions: Synthesizing Information to Support Transcriptomics (RNASSIST), an analytical workflow to use the evident parts of the signal (DEGs) to extract overlooked signatures that are potentially linked to disease phenotypes. RNASSIST is based on the idea that genes do not work in isolation and we can take advantage of the regulatory knowledge provided by a co-expression network to connect each gene with DEGs to understand a potential role of these genes that are not differentially expressed in the disease of interest (Langfelder and Horvath, 2008; Williams, 2015). The input data for RNASSIST include both differential expression analysis (DEA) (Fig. 1A) and co-expression network (Fig. 1B). Using graph embedding (Fig. 1C) and machine learning methods (Fig. 1D), RNASSIST links the structure in the network to differential expression. The extracted features are then used to identify signals relevant to a disease from the network (Fig. 1E).

For the algorithm development, we chose the transcriptomic data from Kapoor *et al.* as it is the largest publicly available transcriptomics dataset for Alcohol Use Disorder (AUD), a psychiatric disorder with small effect sizes (Kapoor *et al.*, 2019). This study utilized RNA-sequencing (RNA-seq) from post-mortem samples of dorsolateral prefrontal cortex (PFC), a region known to be involved in AUD. Importantly, metadata from individuals with recorded alcohol traits including Alcohol Use Disorder Identification Test (AUDIT), drinking years and alcohol drinking per day are available for linking the signals to alcohol drinking behavior. The abundant information in this dataset allowed us to focus on extracting signals based on alcohol traits. Furthermore, given that the |log_2_(fold change)| for this dataset was under 0.4, a commonly used cutoff of |log_2_(fold change)| > 2, no genes would pass the threshold for differential expression. Thus, this dataset was used to demonstrate the power of RNASSIST in finding additional gene candidates for AUD.

RNASSIST identified 829 candidate genes called the critical genes that likely play important roles in AUD that are not necessarily differentially expressed. Of the 829 genes, only 30 were DEGs with adjusted *P*-value < 0.05. This indicates that 799 AUD-related genes would not have been found by existing transcriptomic analysis. In addition, 34 of the critical genes were also the genes that regulate DEG based on database search. Correlations between the critical genes, DEGs and alcohol traits suggest that the identified critical genes are correlated to alcohol traits to the same extent as the top 50 DEGs sorted by |log_2_(fold change)|. Finally, a comparison of the critical genes identified in this study with previously identified alcohol-related genes from mice showed that the critical genes and the neighbor genes overlap with known alcohol genes in mice, whereas human DEGs had no overlap. We believe that this finding reflects the inherent reproducibility limitations of common transcriptomics analysis approaches. As Łabaj and Kreil (2016) shows that the percentage of genes identified as differentially expressed in the same direction could be as low as 20% when different sequencing sites and algorithms for mapping the reads were used. These observed results indicate that the candidate genes overlooked by the standard transcriptomic analyses could be captured by RNASSIST.

## 2 Materials and methods

### 2.1 Differential expression analysis (DEA) and WGCNA network analysis

We obtained the differential expression analysis and the Weighted correlation network analysis (WGCNA) network data directly from Kapoor who published the results and the detailed analyses can be found in the study by Kapoor *et al.* (2019). Briefly, the DEA was done by filtering out the low expression genes and adjusted for the chosen covariates including DSM4 alcohol classification + sex + age + PMI + RIN + batch.

The network was constructed with the WGCNA package in R (Langfelder and Horvath, 2008) and the network modules were determined by hierarchical clustering with the minimum module size = 100, cutting height = 0.99, deepSplit = TRUE. The modules were merged if they the eigengenes had correlation >75%.

### 2.2 Network embedding

The Topological Overlap Matrix (TOM) network output from the WGCNA from Kapoor *et al.* was embedded with GGVec with n_components = 64, order = 1, max_epoch = 100, learning_rate = 0.1, negative_ratio = 0.15 and total_samples = 75 (Ranger, 2021).

### 2.3 Percentage of significant genes per module/cluster for each alcohol trait group

AUD and the matched control samples were manually grouped for each alcohol trait to measure the percentage of genes that were significantly different across the alcohol trait groups. The samples were grouped into 4 AUDIT classes: below 25, between 25 and 50, between 50 and 100 and above 100. AUDIT is a standard tool to screen for alcohol abuse during the past year (Babor *et al.*, 2001). The alcohol intake per day was divided into 4 classes: under 50, between 50 and 100, between 100 and 300 and above 300. The drinking years were also grouped into 4 classes: under 20, between 20 and 30, between 30 and 40 and above 40. All of the above classes were determined so each class had approximately equal number of samples.

For each module/cluster, we evaluated if the gene expression was different among the alcohol trait group. For example, we determined if the genes in each module were statistically different among the four AUDIT classes by one-way ANOVA. The percentage of the genes that were significant was then calculated. The same procedure was repeated for alcohol intake per day and drinking years.

### 2.4 Module/cluster and alcohol trait correlation

The method is the same as the moduleEigengenes() and cor() in the WGCNA library that quantifies module-trait associations (Langfelder and Horvath, 2008). We implemented the method in Python. In brief, eigengene expression for each module/cluster was obtained using the first principal component of all the genes in that module/cluster. The correlation of the eigengene with each alcohol trait was then calculated by Pearson correlation.

### 2.5 Critical genes/neighbor genes/DEGs and alcohol trait correlation

Similar to the ‘Module/cluster and alcohol trait correlation’ described above, the correlation for the genes were simply the correlations of critical gene expression, neighbor gene expression or DEG expression with each alcohol trait calculated by Pearson correlation.

### 2.6 Machine learning to predict each gene as an impact gene or a non-impact gene

The genes were labeled as impact if the |log_2_(fold change)| is in the top 8% of all the differential expressions in the data and the rest would be non-impact. 8% in the Kapoor *et al*.’s data was |log_2_(fold change)| of 0.1. Once the genes were labeled, non-impact genes were downsampled to be equal in number as the impact genes to obtain a balanced dataset. We used max_iter = 1000 for Logistic regression (LR) with other settings as default. Random forest classifier (RF) and XGBoost classifier (XGB) were used with default settings. LR and RF were implemented through the scikit-learn library and XGB was implemented through XGBoost Python package. The balanced data were split into 80% for training and 20% for testing. The feature importance was the model coefficient for LR and the feature_importances_ attribute for RF and XGB. Each model was repeated three times.

### 2.7 Critical gene identification algorithm

***Input***: G = list of impact genes, E = network embedding matrix (gene x feature), D_*k*_ = a list of important dimensions determined by the model *k, for k*ϵ{1,…M}, σ = distance cutoff

***Output***: CG_count = a list of critical genes with counts of nearby impact genes

For *k* in {1, … M}

E_k_ = E [:, D_k_]   (embedding with only the important dimensions)

S_k_ = Euclidean(e_i_, e_j_) (pairwise Euclidean similarity on all the genes (rows) of E_k_)

S_I_ = S_k_ [:, G]   (keep only the distance between genes and impact genes)

CG[:, *k*] = count (S_I_ < σ) (count number of impact genes within the distance cutoff)

CG_count = sort($\sum_{i=1}^{M}CG$[:, i]) in descending order (sum and sort counts for all models)

Output CG_count

### 2.8 Neighbor gene identification algorithm

***Input***: G = impact genes, T = TOM network adjacency matrix (gene x gene), ɛ = edge weight cutoff

***Output***: Neighbor_count = a list of neighbor genes with counts of nearby impact genes

T_I_ = T [:, G]   (keep only the TOM between genes and impact genes)

N = count (T_I_ > ɛ)   (count number of impact genes within the cutoff)

Neighbor_count = sort (N) in descending order (sort counts)

Output Neighbor_count

## 3 Results

### 3.1 Workflow of an RNASSIST to identify critical genes

**Fig. 2.**
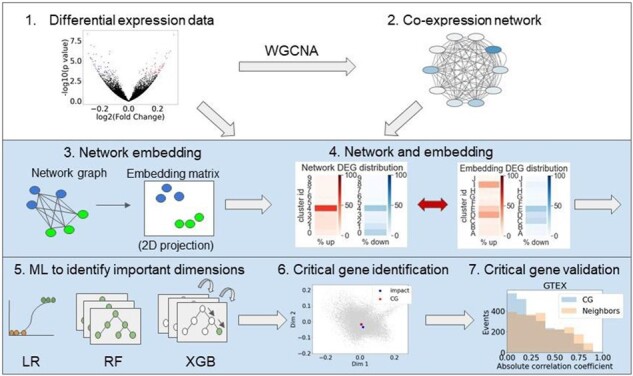
Workflow of RNASSIST to extract critical genes. RNASSIST workflow takes differential expression data (Step 1) and its co-expression network (Step 2) as inputs. RNASSIST (shaded blue) uses network embedding to convert the network to a matrix representation of the network (Step 3). Embedding quality is checked by comparing the network and embedding to ensure that the embedding preserves information from the network (Step 4). RNASSIST then trains three ML models (LR, RF and XGB) to determine the importance of each dimension in the embedding (Step 5), identifies critical genes using the important dimensions based on the distance to impact genes (DEGs with a higher threshold) (Step 6) and validates the roles of critical genes in AUD (Step 7)

The RNASSIST workflow is shown in Figure 2. The preprocessing steps for RNASSIST include DEA (step 1) and co-expression network construction by the WGCNA package (step 2) (Langfelder and Horvath, 2008) from transcriptomic data. RNASSIST (shaded blue) then takes outputs from steps 1 and 2 as inputs and uses a graph embedding to convert the co-expression network to a 64-dimensional vector representation of the network (step 3). RNASSIST compares the network and embedding to ensure that the embedding preserves information from the network (step 4), trains three machine learning (ML) models: logistic regression (LR), random forest classifier (RF) and XGBoost classifier (XGB) to determine the importance of each dimension in the embedding (step 5) (Breiman, 2001; Chen and Guestrin, 2016; McCullagh, 1984), identifies critical genes using the important dimensions found in the previous step (step 6) and, validates the roles of critical genes in AUD (step 7).

### 3.2 Comparison of embedding clusters to network modules validate that the embedding preserves biological meaning from the network

ML requires its input to be vectorized so before applying an ML model on the network, we converted the network to a 64-dimensional vector representation called an embedding. As a network graph and its embedding are structurally different, we compare the network modules and the embedding clusters as a proxy for comparing the network and the embedding to ensure that the embedding preserves information from the network. A network module is a group of genes clustered together based on connectivity while an embedding cluster is a group of genes clustered together based on distance in the embedding. If the embedding preserves a significant amount of information from the network, the embedding clusters should resemble the network modules.

We first performed network module detection by hierarchical clustering (Supplementary Fig. S1A) and Louvain algorithm (Supplementary Fig. S1B). The hierarchical clustering is the module detection method in the WGCNA package, also a standard approach for module detection for a co-expression network. Hierarchical clustering is sensitive to the height where the dendrogram is cut and has a time complexity of *O*(n^2^) (Langfelder and Horvath, 2012). In contrast, Louvain modularity for the module detection has a computational cost of *O*(nlog n) and the module assignment did not change as dramatically when different parameters were used (Blondel *et al.*, 2008). We chose to use Louvain for the module detection method for this dataset. Next, we performed embedding on the whole network using an ultrafast embedding algorithm (Ranger, 2021). Once computed, the genes in the 64-dimensional embedding are clustered by k-means clustering. We used the number of modules detected by the Louvain algorithm as the k for k-means which will allow for direct comparison between the modules and clusters. In the embedding, the genes are a lot more evenly distributed among the clusters than in the modules (Supplementary Fig. S1B and C). This suggests that the embedding connects the genes more effectively than in the network.

**Fig. 3.**
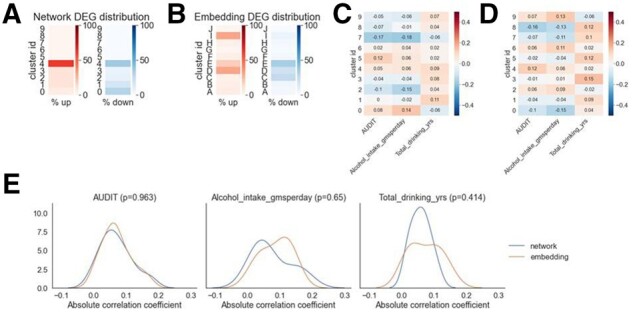
Comparison of network modules and embedding clusters found that the network embedding preserves the biological contexts from the network. (**A**) Percentage of DEGs in each network module. (**B**) Percentage of DEGs in each embedding cluster. The embedding preserves information about the network and so the majority of the DEGs are kept in a few subsets of clusters. (**C**) Percentage of genes in each module that are significantly different by alcohol trait categories. The significance was determined by one-way ANOVA. (**D**) Percentage of genes in each cluster that are significantly different by alcohol trait categories. (**E**) Kernel density estimate (KDE) plots to show the percentage of significant genes for each alcohol trait between the network modules and embedding clusters from C and D. *P*-values are determined by two-sided t-test statistics

Next, we used the biological context such as correlation with alcohol traits to determine whether the embedding preserved relevant information from the network. We compared the percentage of differentially expressed genes (DEGs) in each network module versus the percentage of DEGs in each embedding cluster. DEGs were defined by having adjusted *P*-values < 0.05 by DESeq2 analysis (Love *et al.*, 2014). The majority of DEGs aggregated into one network module (Fig. 3A), whereas the DEGs aggregated into three embedding clusters (Fig. 3B). This analysis informed us that the embedding retained information from the network by keeping the majority of the DEGs in a small subset of the clusters.

Another way to evaluate the embedding quality is to determine the correlation between the alcohol traits and the eigengenes in each network module/embedding cluster (Langfelder and Horvath, 2008). Eigengenes are the first principal component of the gene expression in each network module, a standard feature used in the WGCNA package to measure module and trait correlation. The Pearson correlation is calculated on the eigengenes and the values of each alcohol trait (Fig. 3C and D). To quantify the similarity of the cluster and trait correlation, the correlation coefficients from all the network modules and the embedding clusters are compared and the statistical differences are calculated with t-tests. None of the alcohol traits show statistical significance (*P* < 0.05) between the network modules and the embedding clusters (Fig. 3E). Based on these measures, we determined that the embedding provides us a biologically relevant representation of the network that can be used to find critical genes.

We also determined if the embedding maintains similar patterns for their correlation with alcohol-related traits as in the network data. This is done by grouping the samples based on their traits including the AUDIT score category, the alcohol intake category and drinking years (see Section 2 for details). We then calculated the percentage of genes in a module that were significantly different across the four AUDIT groups (Supplementary Fig. S2A). We repeated the same method for the embedding clusters (Supplementary Fig. S2B). To quantify if the percentage of significant genes are statistically different between the network modules and the embedding clusters, we performed *t*-tests between all the modules/clusters for each trait. None of the traits shows a significant difference between the modules and clusters (Supplementary Fig. S2C). The result confirms that the embedding could represent the network for ML.

### 3.3 ML To extract important dimensions and identify critical genes

We sought to use ML to predict if a gene is an impact gene to identify the features that contributed to distinguishing between DEGs and non-DEGs. An impact gene is defined as a DEG with |log_2_(fold change)| > 0.1. We set this threshold for two reasons: first, most of the genes had small |log_2_(fold change)|. Differentiation between these genes as DEGs and non-DEGs would be challenging without a threshold. Second, this threshold is in the 90th percentile of the fold changes that make up the DEGs and is more likely to extract features that are more relevant to AUD than a lower threshold.

**Fig. 4.**
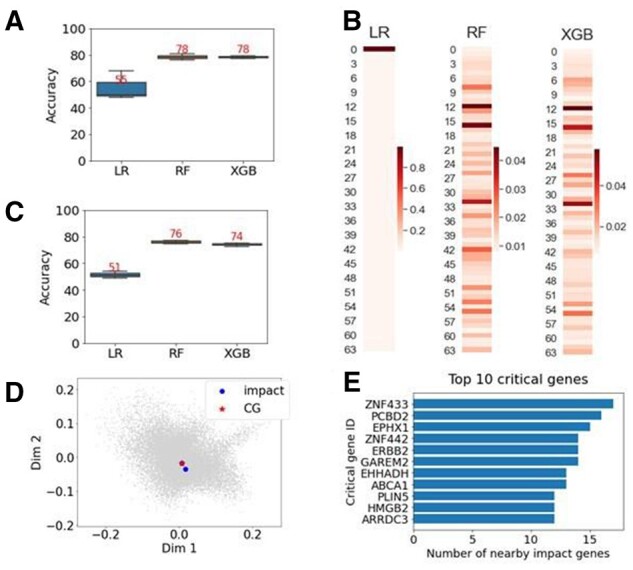
Use of ML to predict impact or non-impact genes in the embedding as a way to extract the important features from the dimensions. (**A**) Accuracy of three ML models: LR, RF and XGB with RF and XGB performed significantly better than LR. (**B**) Heatmaps show the importance of the features (dimensions) in the 64 dimensions of the embedding for each model. Importance is measured by the dimension’s contribution to the predictive performance of the model. (**C**) To validate that the top dimensions are truly critical for the model, the top dimensions that make up >50% of the predictive capability of the model were used to train and test the same ML models (compare with A). (**D**) As a means of ‘objective-driven dimensionality reduction,’ the top dimensions are used to identify critical genes, which are genes that are close to impact genes from the perspective of the ML model. Impact genes are the top 8% differentially expressed genes. For this dataset, 8% is |log_2_ (fold change)| > 0.1. A 2D scatter plot using the two most important dimensions identified by ML is shown as an example. The red star is a critical gene because it is close to multiple impact genes in the most important dimensions. Only two dimensions are shown but the top dimensions with importance sum up to 50% are used for critical gene identification. (**E**) The critical genes are ranked by the number of nearby impact genes. A critical gene is considered to play an important role if it is near multiple impact genes. Top 10 critical genes are shown

The goal of using ML is to integrate the DEA and network analysis to extract the most important embedding dimensions that discriminate between the impact genes and non-impact genes. We trained three ML models to predict each gene as impact or non-impact. RF and XGB both achieved 78% accuracy while LR achieved 55% in accuracy (Fig. 4A). These models were chosen because they are explainable and readily provide the contribution of each feature (dimension) to the model’s performance. The importance of each of the 64 dimensions in the embedding was provided as the model outputs (Fig. 4B). We then ranked the dimensions by the importance from highest to lowest and took the top dimensions that summed up to at least 50% of the total importance. These dimensions are then defined as ‘top dimensions’ and used in the downstream analyses. To determine if the threshold of 50% of total importance is truly crucial for the learning, we used only the top dimensions to train and test the same ML models. We then compared the accuracy of using only the top dimensions in each of the three ML models to using the 64 dimensions. The best accuracies ranged between 93% and 97% of the original accuracy scores so were sufficient to capture the majority of the features the models used (Fig. 4C). Once the important dimensions were found, critical genes were identified based on their proximity to the impact genes in the important dimensions by Euclidean distances (see Section 2 for details). For visualization purposes, only the two most important dimensions are shown (Fig. 4D). A critical gene (red) was identified because it was close to multiple impact genes (blue) in these dimensions. The top ten critical genes are shown in Figure 4E.

### 3.4 Validation of the AUD role of the critical genes

**Fig. 5.**
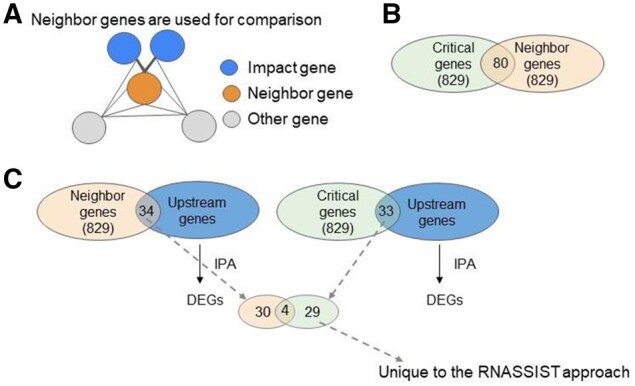
The link between AUD and the critical genes is validated by IPA to be potential upstream regulators of the DEGs in this dataset. (**A**) A neighbor gene (orange) is identified if it is close to several impact genes (blue). ‘Close’ is defined by having the edge weight that connects the neighbor gene and the DEGs above a specific cutoff (see Section 2 for detail). The cutoff was set at a value so that the number of neighbor genes would be equal to the number of critical genes. (**B**) 80 of the critical genes were also neighbor genes. (**C**) We used IPA to find the genes that regulate DEGs and call them the DEG regulators. Then, we determined how many of the neighbor genes and the critical genes were also these DEG regulators. 34 neighbor genes (orange) and 33 critical genes (green) were also DEG regulators. Of the 34 and 33 genes, only 4 were in common. 29 of the critical genes which were DEG regulators would not have been found in this transcriptomic dataset by standard analysis

Gene co-expression can be used to identify regulatory roles (van Dam *et al.*, 2018). Thus, to validate that the identified critical genes indeed play a role in AUD, we compared them with a separate list of genes called the neighbor genes which will be treated as the positive control. The neighbor genes are directly connected to the DEGs in the co-expression network (Fig. 5A) and do not need ML to be identified. We hypothesized that these DEG neighbors which share strong co-expression patterns with the DEGs would likely play a role in AUD or regulate AUD genes. We set a weight cutoff to get the number of neighbor genes that would be equal to the number of critical genes. This provided a list of 829 genes for each gene list. 80 of the critical genes were also neighbor genes, so each gene list had 749 unique genes (Fig. 5B). Ingenuity Pathway Analysis (IPA) was used to find regulators of DEGs. We then determined how many of the critical genes/neighbor genes are these DEG regulators. Thirty-four of the neighbor genes were DEG regulators and 33 of the critical genes were DEG regulators (Fig. 5C). Between the 34 and 33 regulators, only 4 overlapped. This means that 29 critical genes that were also DEG regulators would not be found by a different approach. To evaluate if getting 33 or more DEG regulators from a list of 829 genes was due to a random chance, we performed a hypergeometric test and found that the probability of having 33 or more DEG regulators was < 0.03, meaning that it was unlikely the observed number of overlap was due to random chance.

### 3.5 Validation of link between AUD and critical genes with two complementary datasets

**Fig. 6.**
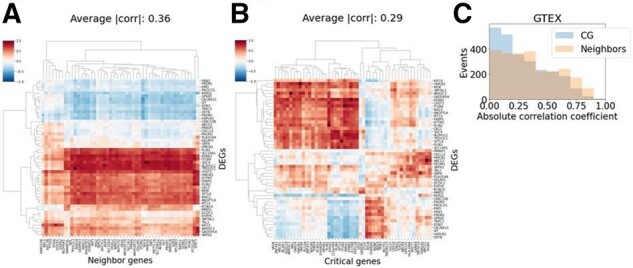
The critical genes share similar correlation with the DEGs as the neighbor genes in an independent and complementary data source (GTEx v8 brain frontal cortex mRNA). (**A**) The correlation heatmap to show the relationship between the top 50 neighbor genes (*x*-axis) and the top 50 DEGs (*y*-axis). The average absolute correlation coefficient is 0.36. (**B**) The expression correlation between the top 50 critical genes (*x*-axis) and the top 50 DEGs (*y*-axis). The average absolute correlation coefficient is 0.29. (**C**) Overlaying all the absolute correlation coefficients for the critical genes (CG) and the neighbors show that the critical genes are similarly correlated to the AUD DEGs as the neighbors. For calculating the statistics, the absolute correlation coefficients were used instead of the raw coefficient values because the goal was to compare the size of the correlation not the direction of the correlation. Two-sided t-test determined that the correlation coefficients were statistically insignificant with a *P*-value = 0.275

We also validated the link between AUD and the critical genes using two complementary datasets that are independent of the one used to discover the critical genes. We first used the Genotype-Tissue Expression (GTEx) v8 frontal cortex mRNA resource which represents another post-mortem brain dataset with 209 subjects (Aguet *et al.*, 2020). The purpose of using a separate but related dataset is to show that if we can find co-expression patterns of the critical genes and AUD DEGs, it would suggest that the critical genes are likely to regulate or be regulated by DEGs. If these critical genes regulate AUD DEGs, then they are likely to play a role in AUD. The subjects for the frontal cortex samples in GTEx had no specific causes of death and were distributed across age groups and both sexes (Supplementary Fig. S3). We found that the average absolute correlation coefficients between the neighbor genes and the DEGs were 0.36 (Fig. 6A). For the critical genes, it was 0.29 (Fig. 6B). As expected, the neighbors of the DEGs discovered in the co-expression network were more correlated than the critical genes. However, when we overlaid the absolute correlation coefficients from these two comparisons, there was no statistical significance between them (Fig. 6C). Despite the neighbor genes being the apparent regulatory candidates since they are direct neighbors of the DEGs in our dataset, the critical genes identified by the RNASSIST algorithms were as correlated with the DEGs in the complementary dataset and therefore, gave us more confidence that the critical genes play a role in AUD.

Second, we compared the identified critical genes with genes known to be important for alcohol-related behaviors in mice (Mayfield *et al.*, 2016). Out of 111 mouse genes that share human homologues in the alcohol gene list, 7 are the critical genes, 8 are the neighbor genes and none are the DEGs (Table 1). The results further support that the critical genes identified in our study are likely to be involved in AUD.

**Table 1.** Validation of the role of DEGs, critical genes and neighbor genes by comparing to the previously known alcohol-related genes in mice (Mayfield *et al.*, 2016)

|	Overlap genes	No. of overlap	Hypergeometric *P*-value (The probability of observing # or more overlap due to random random)
Known mouse alcohol-related genes versus DEGs	None	0	1
Known mouse alcohol-related genes versus critical genes	CHRNA4, CHRNA6, CRHR2, FYN, GRM4, TLR2, TRPV1	7	0.092
Known mouse alcohol-related genes versus neighbors	ADIPOR2, AGT, CHRNA6, GABRA1, MAOA, NTSR2, SLC1A3, TGFA	8	0.042

*Note*: The hypergeometric test was used to determine the probability of observing the number or more overlap. A small probability (*P*-value) means it is unlikely the observation is due to random chance.

### 3.6 Validation of AUD role of the critical genes based on correlation with alcohol traits

We also measured the correlation between the expression levels of the critical genes and the alcohol traits to determine the involvement of the critical genes in AUD. The DEGs and the neighbor genes are the apparent signals so they serve as the positive control groups. Interestingly, we found the correlation patterns are similar among the critical gene and the DEG groups (Supplementary Fig. S4A and C). We quantified the difference in their absolute correlation coefficients with one-way ANOVA and found that the DEGs and the critical genes have no statistical difference in terms of their correlation to alcohol traits (Supplementary Fig. S4D). Only the neighbor genes showed statistically higher correlation coefficients than the other two gene lists for AUDIT (adjusted *P*-values < 0.001). These results emphasize that although 96.4% of the critical genes are non-DEGs, they show a similar level of correlation with the alcohol traits as DEGs.

### 3.7 Validation of robustness of the RNASSIST algorithm on a second dataset

To test the robustness of the RNASSIST algorithm, we applied the same workflow to an independent publicly available dataset which were microarrays that compared the PFC of binge-like drinking mice and the controls (Ferguson *et al.*, 2019). Alcohol traits for this dataset were not available because the mice were ethanol-naive so the workflow was applied by removing all the steps with alcohol traits involved. We found that the embedding preserved significant information from the network as the percentage of DEGs aggregated into a similar number of clusters of the embedding as in the modules of the network (Supplementary Fig. S5A and B). ML achieved 86-88% of accuracy for the three models (Supplementary Fig. S5C). By comparing to the previous literature, critical genes had the most overlap with the known alcohol-related genes than DEGs and the neighbor genes in this dataset (Supplementary Table S1). This confirmed that the RNASSIST algorithm not only works well with RNA-seq data, but also with microarray data across species.

## 4 Discussion

RNASSIST uses ML models to find features to connect genes to DEGs in a transcriptomic dataset and identifies a list of critical genes that likely play important roles in disease under investigation. Here, RNASSIST found 829 AUD critical genes with only 30 of them being differentially expressed. Through various validation approaches, we determined that it was likely that these critical genes regulate and co-express with DEGs. Many of these critical genes would likely be missed by standard transcriptomic analyses. Note that, the identified critical genes can be either DEGs or non-DEGs as long as they are the closest neighbors of impact genes in the important dimensions. Researchers may combine the top DEGs and the top critical genes for downstream analysis to ensure a maximal number of signals are considered.

For the network embedding approach, we used an ultrafast embedding method to vectorize the entire co-expression network that had 19911 nodes (genes) with almost 4 million edges. Since it is typical for an organism to have tens of thousands of expressed genes, the ultrafast embedding is considered more appropriate than techniques such as DeepWalk or Node2Vec (Grover and Leskovec, 2016; Perozzi *et al.*, 2014). We originally used Node2Vec as our network embedding method as it has parameters to balance between breadth-first search and depth-first search for its random walks. However, using Node2Vec to embed a network with millions of edges was too computationally intensive (∼40 minutes for a network with 10000 edges using 32 GB RAM Intel Core i7-1065G7 CPU 4 cores). We tried sub-selecting the whole network and embedded individual subnetworks with Node2Vec. This led to issues on how to best sub-select the network to ensure the information lost during the network sub-selection process was negligible. As a result, we decided to use the ultra-fast embedding method called GGVec (Ranger, 2021). How GGVec could achieve rapid embedding is beyond the scope of this article but we found that embedding the whole network did preserve more information than when the network was sub-selected and then embedded.

In a WGCNA network, the local information would be the co-expression patterns of the genes and their neighbors. One way to describe the global structure of the network is to look at the network modules, which are treated as gene sets enriched with functions or pathways (Langfelder and Horvath, 2016). When the network is converted to the embedding, both the local and the global structures are taken into account (Ma *et al.*, 2018; Nelson *et al.*, 2019). As a result, we believe the features in the embedding encode information to preserve the co-expression patterns and the biological functions of the network. However, what the important dimensions extracted by the ML models that were used to find critical genes means biologically is unclear at this point. This would be an interesting question to explore in the future.

RNASSIST used an approach different from a standard transcriptomic analysis workflow to identify additional AUD candidates. However, RNASSIST does not replace the existing transcriptomic analysis. The algorithm depends on normalized expression and DEA as inputs. Identification of both the neighbor genes and the critical genes rely on the knowledge of DEGs in the dataset discovered by the standard transcriptomic analysis. The main goal of RNASSIST is to extract as many signals as possible from a high-throughput dataset rather than replacing the existing methods. We summarized the main differences between the standard transcriptomic workflows and RNASSIST in Table 2. In short, RNASSIST removes places where prior knowledge and subjective decisions are required and allows an unbiased method to discover unknown signals.

**Table 2.** RNASSIST removes places where prior knowledge and subjective decisions are required to identify candidate genes

|	Candidates	Need prior Knowledge for module functional annotation	Manual cutoff for network modules
Commonly used approach: DEGs + network modules with enriched DEGs	Restricted mostly to DEGs	Yes	Yes
RNASSIST	Not restricted to DEGs	No	No

*Note*: ‘Candidates’: top candidates found by the algorithm. ‘Need prior knowledge for module functional annotation’: this means that a common approach requires existing knowledge to functionally annotate the modules before determining if a module is relevant. If a module fails to meet that criterion, even if it could play a role in the disease, it is often ignored. ‘Manual cutoff for network modules’: the height where the dendrogram is cut is a manual decision and this decision could affect if a gene belongs to a functionally relevant module or not.

The DEA for the two transcriptomic datasets used in this study were analyzed with DESeq2 and limma, respectively (Love *et al.*, 2014; Ritchie *et al.*, 2015). Comparison of DEA tools has shown that different tools will likely yield different sets of DEGs with overlap between them. The extent of the overlap depends on which tools are being compared (Costa-Silva *et al.*, 2017; Seyednasrollah *et al.*, 2015; Zhang *et al.*, 2014). Since RNASSIST relies on impact genes, DEGs with a higher |log_2_(fold change)| cutoff, to find other signals, it is likely that these impact genes would be shared across DEA tools. We recommend that if a DEA method is known to have better performance on the tissue or organism researchers work with, using that tool would likely be beneficial to identify the critical genes as RNASSIST depends on the DEGs to link other signals.

In summary, this work not only provides direct impact to the alcohol research community by finding AUD-related genes that would not otherwise be found, but also demonstrates the power of a new approach to identify hidden signals in a large transcriptomic dataset that is applicable to all transcriptomic work.

## Supplementary Material

btab673_Supplementary_Data
