# Supplementary material for: RNA Solutions: Synthesizing Information to Support Transcriptomics (RNASSIST)
Source: Bioinformatics. 2021 Sep 27;38(2):397–403. doi: 10.1093/bioinformatics/btab673 (PMC8723147; doi:10.1093/bioinformatics/btab673)
Supplement: btab673_Supplementary_Data [file btab673_supplementary_data.zip › Supplementary Table 1.docx]

|  | Overlap genes | # overlap | Hypergeometric p-value (The probability of observing # or more overlap due to random chance) |
| --- | --- | --- | --- |
| Known mouse alcohol-related genes vs mouse DEGs | Ntsr1 | 1 | 0.236 |
| Known mouse alcohol-related genes vs mouse critical genes | Ctsf, Pdyn, Prkg2 | 3 | 0.081 |
| Known mouse alcohol-related genes vs mouse neighbors | Grin2a, Rab3a | 2 | 0.226 |
