# Supplementary figures and images for: RNA Solutions: Synthesizing Information to Support Transcriptomics (RNASSIST)
Source: Bioinformatics. 2021 Sep 27;38(2):397–403. doi: 10.1093/bioinformatics/btab673 (PMC8723147; doi:10.1093/bioinformatics/btab673)

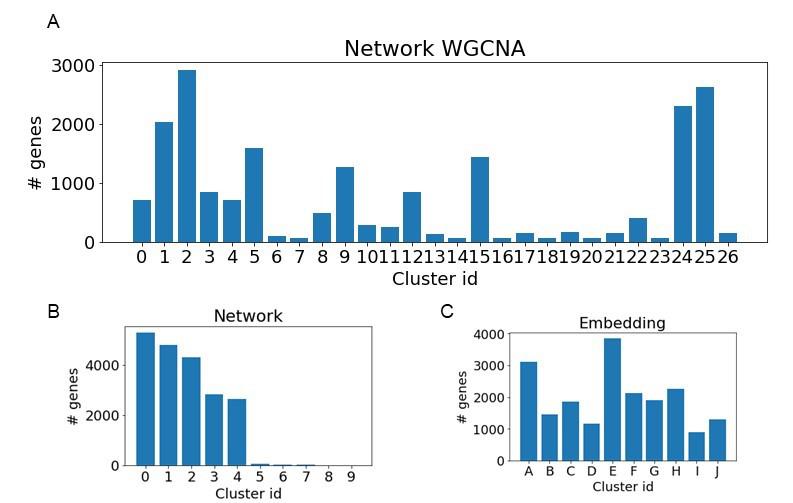

Supplement: btab673_Supplementary_Data [file btab673_supplementary_data.zip › 350_Supplementary Figure 1.JPG]

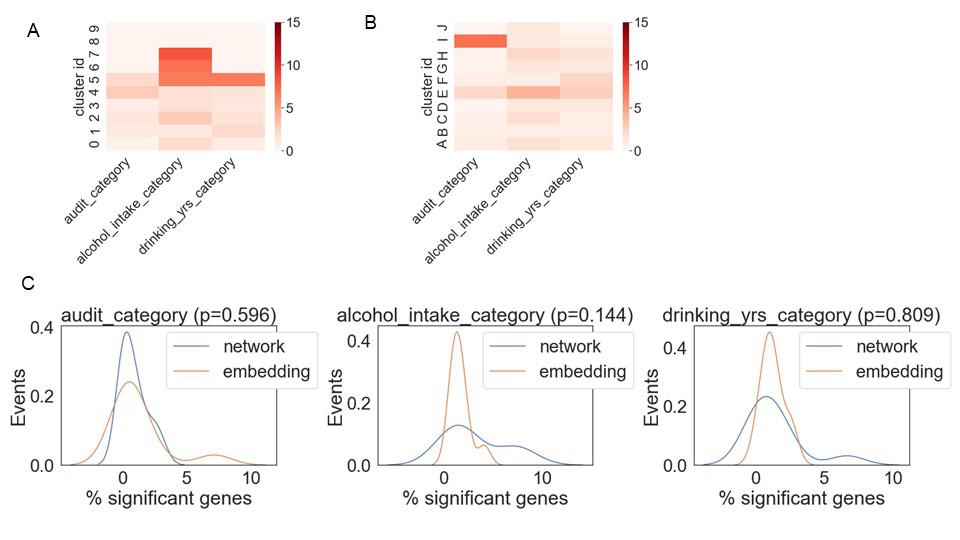

Supplement: btab673_Supplementary_Data [file btab673_supplementary_data.zip › 350_Supplementary Figure 2.JPG]

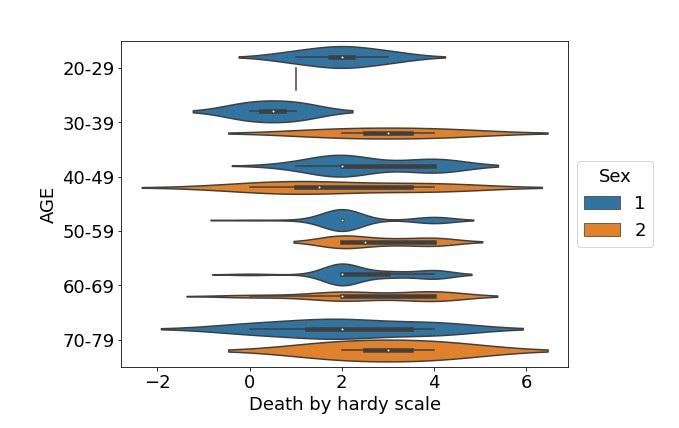

Supplement: btab673_Supplementary_Data [file btab673_supplementary_data.zip › 350_Supplementary Figure 3.JPG]

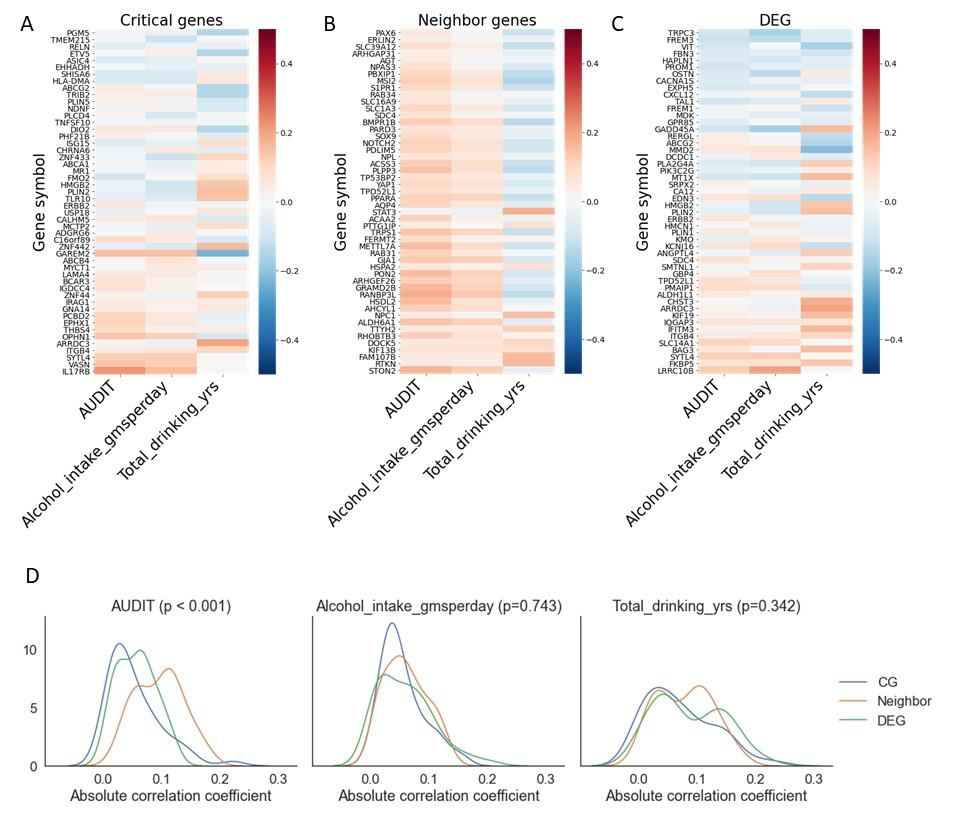

Supplement: btab673_Supplementary_Data [file btab673_supplementary_data.zip › 350_Supplementary Figure 4.jpg]

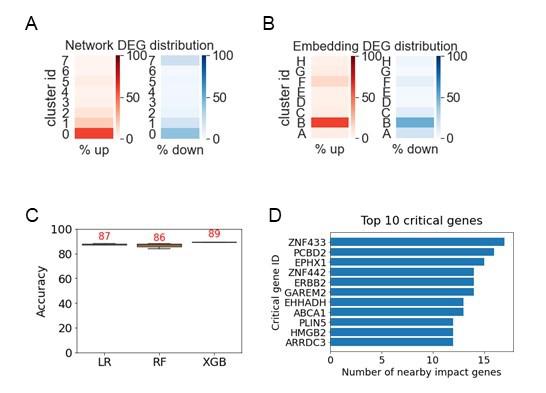

Supplement: btab673_Supplementary_Data [file btab673_supplementary_data.zip › 350_Supplementary Figure 5.JPG]
